# Infanticide in London

**Published:** 1864-11

**Authors:** 


Infanticide in London.—Official reports show that, during the year 1861, there were held 1,103 inquests on children who had died violent deaths.

